# Utilizing Deep Learning for Defect Inspection in Hand Tool Assembly

**DOI:** 10.3390/s24113635

**Published:** 2024-06-04

**Authors:** Hong-Dar Lin, Cheng-Kai Jheng, Chou-Hsien Lin, Hung-Tso Chang

**Affiliations:** 1 Department of Industrial Engineering and Management, Chaoyang University of Technology, Taichung 413310, Taiwan; alex_cheng2@unimicron.com (C.-K.J.); hch626.cool@gmail.com (H.-T.C.); 2 Department of Civil, Architectural, and Environmental Engineering, The University of Texas at Austin, Austin, TX 78712-0273, USA; chslin@utexas.edu

**Keywords:** hand tools, assembly defects, visual inspection, deep learning, R-CNN series models

## Abstract

The integrity of product assembly in the precision assembly industry significantly influences the quality of the final products. During the assembly process, products may acquire assembly defects due to personnel oversight. A severe assembly defect could impair the product’s normal function and potentially cause loss of life or property for the user. For workpiece defect inspection, there is limited discussion on the simultaneous detection of the primary kinds of assembly anomaly (missing parts, misplaced parts, foreign objects, and extra parts). However, these assembly anomalies account for most customer complaints in the traditional hand tool industry. This is because no equipment can comprehensively inspect major assembly defects, and inspections rely solely on professionals using simple tools and their own experience. Thus, this study proposes an automated visual inspection system to achieve defect inspection in hand tool assembly. This study samples the work-in-process from three assembly stations in the ratchet wrench assembly process; an investigation of 28 common assembly defect types is presented, covering the 4 kinds of assembly anomaly in the assembly operation; also, this study captures sample images of various assembly defects for the experiments. First, the captured images are filtered to eliminate surface reflection noise from the workpiece; then, a circular mask is given at the assembly position to extract the ROI area; next, the filtered ROI images are used to create a defect-type label set using manual annotation; after this, the R-CNN series network models are applied to object feature extraction and classification; finally, they are compared with other object detection models to identify which inspection model has the better performance. The experimental results show that, if each station uses the best model for defect inspection, it can effectively detect and classify defects. The average defect detection rate (1-β) of each station is 92.64%, the average misjudgment rate (α) is 6.68%, and the average correct classification rate (CR) is 88.03%.

## 1. Introduction

For the assembly of industrial products, the industry develops automatic and manual assembly services, which can realize assembly processes of different complexities and meet the processing needs of customers for mass production and customized production. Assembly skills are one of the factors that affect quality. The personnel stationed on the production line have many years of assembly experience and complete personnel training. Appearance defects can be checked simultaneously during assembly to further ensure product quality. Product assembly integrity affects the quality of the final product. If the assembly defects are severe, not only will the product not be able to function correctly, but it may even cause loss of life or property for the user. Therefore, the assembly quality of the product needs to be strictly controlled [1,2].

This study takes the assembly process of the wrench, an essential hand tool, as an example to explore the automated detection of hand tool assembly defects in the precision assembly process. A wrench is assembled from multiple parts, and the parts’ design, manufacture, and assembly are crucial. No matter how well designed the components are, they will not work properly if they are not assembled correctly. As far as the hand tool industry is concerned, wrenches are a standard tool. Ratchet wrenches are currently the most widely used screw-tightening tools. They can be used with sockets of different specifications. When in use, there is no need to adjust the settings again after tightening the screws, which is very convenient in use and improves work efficiency. Since the operation methods of standard ratchet wrench assembly factories are generally based on manual labor, the assembly process is manual processing, and the inspection process is also judged through visual inspection with the human eye. Occasionally, after assembly is completed, wrenches have assembly defects, but the assembly personnel do not discover them through self-inspection and judge them as normal. As a result, the products are returned after being delivered to customers. This phenomenon of missed detection exists at every assembly station. Therefore, this study uses ratchet wrenches as the experimental object to explore and develop an assembly defect detection system for hand tools that require precision assembly.

**Figure 1:**
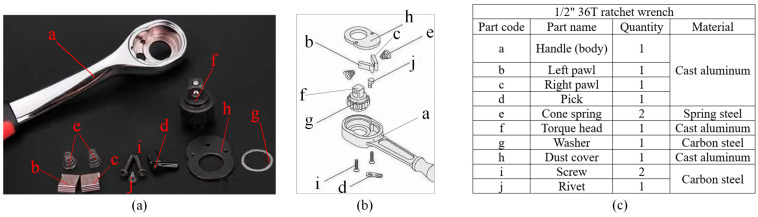
Internal structure of 1/2″ 36T ratchet wrench: (**a**) physical drawing; (**b**) exploded view; (**c**) parts list of assembly parts of the workpiece.

**Figure 2:**
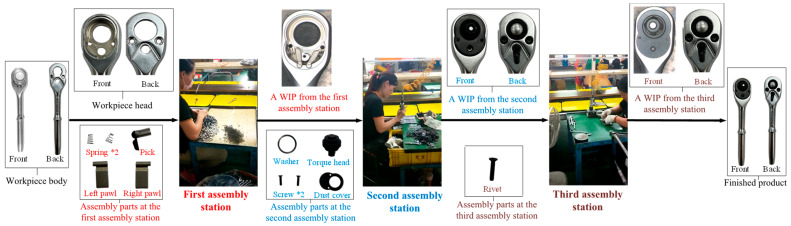
An assembly diagram of the parts required for each assembly station of the ratchet wrench assembly process.

A ratchet wrench is composed of 12 components (10 types). Figure 1 shows (a) the assembly parts diagram, (b) an exploded-view drawing, and (c) a parts list of the ratchet wrench. Various manufacturers have improved their internal structures to create product differentiation. Some manufacturers will change the design of the pawl, and some will change the style of the pick. The research object used in this study is the traditional double-pawl design. The workpiece assembly work of this double-pawl ratchet wrench can be divided into three assembly stations: the body is assembled from the first station, and the left pawl, right pawl, paddle, and spring are installed at the first station; after the assembly, the good WIPs (work-in-process) assembled at the first station are transported to the second station via a conveyor belt; at the second station, the torque heads, washers, dust covers, and screws are installed; after the assembly, the good WIPs assembled at the second station are sent to the third station via the conveyor belt; at the third station, the rivets are installed, and the product assembly process is completed. Figure 2 shows the assembly process and parts collection of each assembly station of the ratchet wrench.

**Figure 3:**
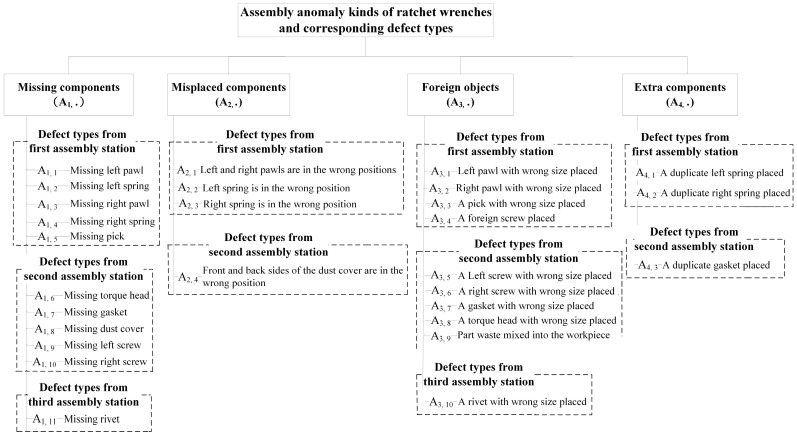
Summary chart of the relationship between assembly anomaly kinds and corresponding defect types of ratchet wrenches.

**Figure 4:**
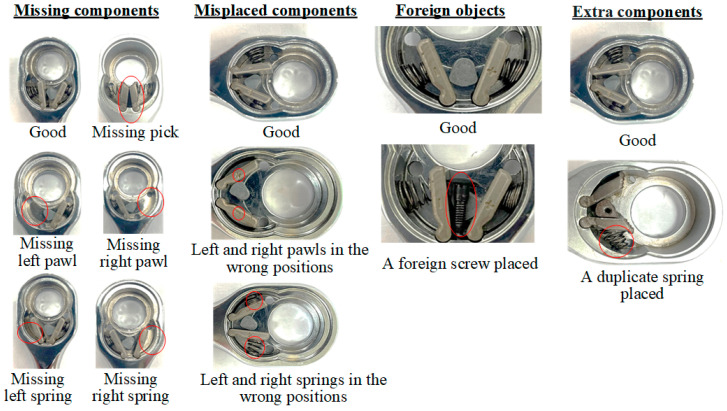
Example images of four kinds of assembly anomalies and corresponding defect types at the first assembly station of the ratchet wrench.

This study explores four assembly anomaly kinds that often occur in general assembly operations: missing components, misplaced components, foreign objects, and extra components. The four kinds of assembly anomalies that may arise during ratchet wrench assembly are very similar in appearance, and misjudgments often occur even with manual visual inspection. Their detailed descriptions and examples are shown in Table 1. These four anomaly kinds can be further subdivided into multiple defect types according to the operation content of each assembly station. Figure 3 shows the relationship between the assembly anomaly kinds that may occur at the ratchet wrench assembly stations and their corresponding defect types. These four kinds of assembly anomalies include nearly 28 types of assembly defects. Historical data show that approximately 35% of total products will have assembly defects under the actual assembly conditions on the production line. Missing parts are the most common assembly anomaly type (60%), followed by misplaced parts (30%), extra parts (5%), and foreign objects (5%). The sequence of product assembly inspection first determines what kind of assembly anomaly the workpiece belongs to and then further determines what type of assembly defect the anomaly belongs to among the four anomaly kinds. Classifying defect types and detecting defect locations are essential for process improvement, allowing process engineers to trace the root cause of problems and take corrective actions. To compare the differences between the types of assembly defects in each assembly station in more detail, Figure 4 shows example images of the four kinds of assembly anomalies and their corresponding defect types that may occur during the assembly process at the first assembly station of the ratchet wrench. In this assembled part set, the screws, springs, and rivets can be used in both the left and right directions, but the left and right pawls cannot be used in both directions due to the different designs of the parts themselves.

The integrity of product assembly affects the final product’s quality. When an assembled WIP enters the assembly process, the parts and assembly modules are assembled manually. If an assembly defect occurs during the manufacturing process (e.g., missing springs or misplaced screws), it could end up in the hands of the customer due to inspection errors, resulting in a potential failure in the finished product. Although human inspectors can provide good visual acuity, their performance can be affected by scene complexity, time constraints, and eye fatigue. Traditional machine vision systems offer speed and repeatability but face challenges with high-dynamic-range imaging, low-contrast scenes, and subtle variations. Although machine learning’s identification of random flaws can be improved through artificial intelligence techniques, this requires many training samples and time. While machine vision systems can adapt to variations in the appearance of a workpiece caused by scaling, rotation, and distortion, the presence of intricate surface textures and image quality problems can pose substantial challenges for inspection [3]. Machine vision systems are fundamentally limited in evaluating the potential for variation and bias among visually similar images. In contrast, computer vision techniques paired with deep learning models can identify defects that other methods miss. These deep-learning-based visual inspection systems excel in identifying defects even in complex assemblies and low-contrast settings. They outperform machine vision in finding random defects and human inspectors in consistency and repeatability [4].

Automated visual inspection systems, powered by deep learning technology, can surpass human inspectors or conventional machine vision systems in performance. Using deep learning and machine vision technology, intelligent systems can be built to conduct comprehensive quality inspections down to the finest details. Deep learning techniques employ neural networks with thousands of layers. These layers emulate human-like intelligence in identifying anomalies, artifacts, and features while accommodating natural variations in intricate patterns. Thus, deep learning merges the versatility of human visual inspection with the rapidity and resilience of computerized systems [4,5]. This study attempts to use this property to extend the deep-learning-based vision system to manual tool assembly inspection, including multiple assembly anomalies and defect types. The kinds of assembly anomalies may contain various parts caused by incorrect assembly, and the defect types caused by the combination of parts become more numerous. The difference in appearance between these types of defects is not apparent. Effectively and efficiently detecting assembly defects is a challenge.

This study’s research method (R-CNN series model) has shown good results in object detection in research into different applications. Still, the literature has not found that it can simultaneously be applied to all assembly anomalies proposed in this study. The development of automated assembly defect inspection of precision parts in the hand tool industry has its difficulties, as detailed here: First, hand tool products are composed of closely clustered industrial components, including fixed-shaped parts (screws, picks, rivets, etc.) and deformable parts (springs, washers, sheets, etc.). After assembly, the appearance of the combined workpiece changes and is variable. Second, a hand tool assembly may combine two or more parts together to produce another new part with a similar appearance. Metal parts will be oily and can easily cause rust due to long-term contact, affecting the appearance of the parts. Third, the assembly embedding process will eliminate the contours of objects that extend into each other’s space in pairs and merge the parts into one entity. Therefore, parts can touch other parts or expand into other parts, and the changes in their shape after assembly are more diverse. Fourth, hand tool parts are made of many different materials. The lighting angle of the auxiliary light source during image capture will cause significantly different reflection effects among various materials, making it difficult for the parts to be imaged in one shot. Fifth, four kinds of assembly anomalies often occur in the ratchet wrench’s assembly operation. Combined with different types of parts, these 4 anomalies cover more than 28 assembly defects. When the appearance differences of the assembled parts are not noticeable, to distinguish so many defect types, it is necessary to have the ability to identify subtle differences. Using traditional machine vision and classification techniques to detect assembled parts with tightly clustered and embedded properties is challenging. This study proposes the R-CNN series models of deep learning technology to identify closely clustered and embedded assemblies, classify assembly defect types, and pinpoint the location of defects.

The subsequent sections of this article are organized as follows: It begins by reviewing the existing literature on the methods currently employed by optical inspection systems for assembly defects of industrial products. Next, we detail the proposed deep learning models to detect assembly defects and determine their locations on the hand tools. This is followed by a series of tests to assess the proposed models’ effectiveness and efficiency, drawing comparisons with conventional techniques. Lastly, we summarize our contributions and suggest potential directions for future research.

## 2. Literature Review

Many modern industries use automated visual inspection for quality inspection. The appropriate application of automated visual inspection technology can effectively reduce the cost of manual inspection [3,4]. Rehmat et al. [6] used automated vision technology to inspect remote control assembly processes related to industrial safety. Huang et al. [7] discussed that visual inspection devices installed in the manufacturing, assembly, and testing processes can quickly and accurately detect abnormalities in the semiconductor process. Ravikumar et al. [8] used computer vision technology to detect surface defects and tool wear abnormalities.

### 2.1. Defect Inspection Methods in the Assembly Process

Most of the current research on defect inspection focuses on inspecting defects on the surface of objects, while this study focuses on inspecting defects during the assembly process of workpieces. Frustaci et al. [9] proposed a machine vision system used in the industrial assembly process to examine the assembly operations of catalytic converters and detect geometric defects caused by plane and rotational displacements. Chen et al. [10] developed a feature-based approach to test the assembly error of the incident angle in the assembly process of turbine blades. Natsagdorj et al. [11] introduced a computer-vision-based golf club head assembly and inspection system. Other related research focuses on precisely positioning the components during assembly [12,13,14,15,16].

### 2.2. Object Detection Models

In object detection, the Region-based Convolutional Neural Network (R-CNN) model was introduced by Ross Girshick [17]; this was the earliest network model to mark the location of objects. Tao et al. [18] proposed a self-driving vehicle detection system using R-CNN to enhance object detection accuracy by minimizing the brightness error. Zhang et al. [19] developed a revised R-CNN mode to find foreign objects that may appear on high-voltage power lines. Huynh et al. [20] applied the R-CNN algorithm to detect whether the bolts in the captured images are loose. The disadvantage of this mode is that the calculation time is too long. The Fast Region with CNN (Fast R-CNN) model was proposed by the original author of R-CNN in response to the problems found in the R-CNN model [21]. Kim et al. [22] used the Fast R-CNN model to improve the situation where human body detection is difficult to achieve at night or in insufficient lighting environments. Bai et al. [23] applied modified Fast R-CNN and optimization functions to effectively improve the detection efficiency of rail fasteners by pre-labeling the data in the dataset. Zheng et al. [24] used the Fast R-CNN method to analyze surface cracks on railway tracks.

Ren et al. [25] introduced the Faster Region with CNN (Faster R-CNN) model by employing the Region Proposal Network (RPN) algorithm to supplant the original selective search, thereby addressing the issue of time-consuming calculations in the Region Proposal. Annapoorna et al. [26] applied Faster R-CNN to find and locate cotton for smaller agricultural products. Zeng et al. [27] incorporated an adversarial occlusion network (AON) with the Faster R-CNN to detect underwater targets in the complexity of the seabed environment and frequent occlusion of objects. Gong et al. [28] used Faster R-CNN combined with geometric imaging theory to find the looseness of threaded fasteners quantitatively. The Mask Region with CNN (Mask R-CNN) model was introduced by He et al. [29]. It was developed in response to the Faster R-CNN issue, where the extracted feature image could not align with the initial ROI. Mask R-CNN is considered the best network in the R-CNN series of models, suitable for large objects and complex backgrounds. Xu et al. [30] applied the Mask R-CNN model combined with feature enhancement to detect tunnel surface defects in complex backgrounds. Zhao et al. [31] combined the Mask R-CNN with a virtual reality wearable device to inspect aviation cable brackets.

YOLOv1 represents the initial application of the one-stage detection method in object detection models [32]. YOLOv4 was proposed by Bochkovskiy et al. [33] in 2020 and is a relatively new model in the YOLO series. Guo et al. [34] proposed an improved version of YOLOv4-hybrid to detect railway defects. Cho et al. [35] compared multiple deep learning network models to detect workpiece objects on conveyor belts. Liu et al. [36] developed a missing part detection method for PCB boards using YOLOv4 and a Gaussian function to merge the prediction frames into the same position to calculate the loss function of the regression frame. This model is also the subject of implementing and comparing the methods proposed in this study.

### 2.3. Assembly Defect Inspection

From the discussion of the above literature, we know that most current defect inspections focus on surface defect inspection. The more commonly discussed surface defects are abnormal changes on the surface of objects, such as holes, cracks, scratches, creases, dirt, labels, or oxidation [8,20,23,24,37,38,39,40], and most product inspections are performed through computer vision inspection at the finished product stage [6,7,8]. This study focuses on the types of assembly abnormalities that may occur in work-in-process (WIP) during the assembly process. Current related research proposed missing parts [9,16,36], misalignment [9,10,11,12,13,14,15,16], remaining parts [41], and foreign objects [19,41]. Most are discussed on individual or specific anomaly kinds [42], and fewer cover multiple anomaly kinds simultaneously. The above four kinds of assembly anomalies have different types of individual defects, and the number of defect types in each assembly anomaly is also different. The inspection method proposed in this study is to conduct the inspection task at each station during the ratchet wrench assembly operation rather than after the product is assembled. This inspection during assembly operations can instantly determine the quality of the work-in-process. If the defect type and location are known, then the cause can be traced immediately, and corrective actions can be taken to achieve the function of prevention in advance.

Based on the four assembly anomalies proposed in this study, up to 28 corresponding defect types can be further analyzed. However, most defect inspection studies do not mention many types of assembly defects, and the characteristics of the research objects in this study are not only similar parts, i.e., small parts with high assembly similarity, but also need to accurately point out the defect locations and classify the defect types. Production line personnel can trace the cause of the abnormality based on the correct defect type and location. Because the parts in precision assemblies are assembled close to each other, traditional image processing technology makes it challenging to process objects that are too tightly clustered. Therefore, this study is expected to apply the algorithms of these R-CNN series models to identify closely clustered components, classify the assembly defect types, and accurately locate the defect occurrences.

## 3. Proposed Methods

This study uses computer vision technology and deep learning models to develop a defect inspection system for ratchet wrench assembly operations. This study first captures the image of each defect type; then, it uses homomorphic filtering to eliminate the reflection generated during the image capture process and captures the inspection area of the workpiece image through the given mask center and radius; then, it uses manual annotation to create an assembly defect-type label. The defect features are then extracted and classified by the label type to which the image belongs. This study initially uses the R-CNN algorithm, and its training can be divided into four steps: Step 1 is candidate area selection, using manual methods to mark the detection area in the input image and using color, size, and texture similarity to separate possible target areas from the detection regions. Step 2 is feature extraction, using the convolution operation in the CNN network to extract image feature values. The current feature extraction part uses five layers of convolutional layers and two layers of fully connected layers for feature extraction. Step 3 is classification, using a Support Vector Machine (SVM) model to score each category individually in the entire feature vector. Step 4 is locating defect location, using bounding box regression to optimize the frame selection area. After the R-CNN network training, the assembly defect identification system is tested, and the identification results are output to create a confusion matrix and evaluate its detection performance.

### 3.1. Image Capture

**Figure 5:**
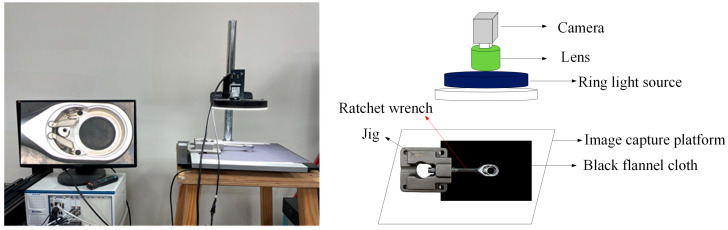
Experimental hardware setup for image capture: a photograph and the corresponding schematic diagram.

First, we need to collect a set of test samples containing examples of the defects the models should learn to identify. All samples were randomly selected from the manufacturing process of a hand tool company in Taiwan. The image acquisition in this study’s preliminary stage was conducted in a laboratory environment. To fix the capturing workpiece during the shooting process, the jig used at this stage is an iron block with length, width, and height of 109.74 mm, 109.52 mm, and 32.31 mm; it has a hole with a diameter of 13.5 mm and a depth of 30 mm, which is drilled at the center point of the width and height so that the handle of the ratchet wrench can insert into the hole. Figure 5 is a schematic diagram of the hardware equipment setup in the early stage of this study.

### 3.2. Image Preprocessing and Image Labeling

**Figure 6:**
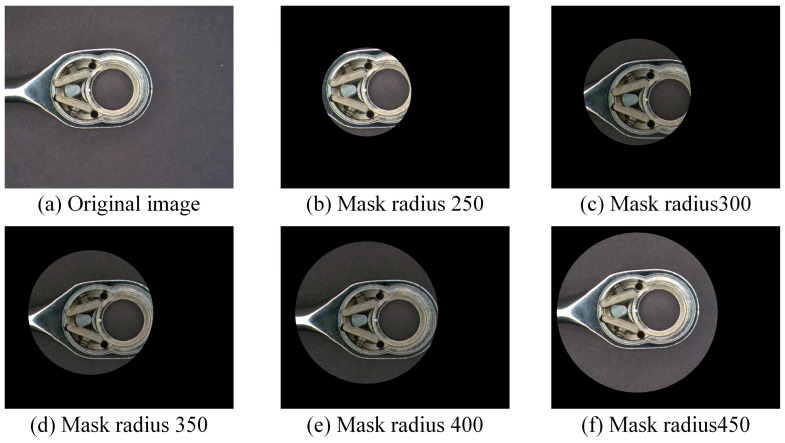
Preprocessed images with five mask radii used in this study (unit: pixel).

First, homomorphic filtering is applied to the captured images to enhance dynamic range compression and contrast. This process helps reduce the impact of reflections during the shooting process [43]. To minimize the impact of the background of the ratchet wrench image on the detection effect, a circular mask is used after the image is captured to eliminate excess parts and leave only the area to be detected. Currently, five mask sizes are used to explore which mask has better detection results; this schematic diagram is shown in Figure 6. The initial image size is 1024 × 1280 pixels, and the mask center point position is (800, 540). Table 2 shows the initially used center radius, the area of the detection area, and the image area ratio occupied by the detection area.

This study will perform feature extraction on the wrench images in the detection area after filtering. In the preliminary stage, the built-in Image Labeler toolbox in MATLAB R2019a marks the position and category of defects. This toolbox only needs to define the name of the detection area label and then load the image for manual marking. If the label is a good product, then the information of the manually annotated bounding box is [427, 379, 208, 326]. The first two pieces of information represent the point in the upper left corner of the manual bounding box, which is also the starting point (*X_i_*, *Y_i_*) = (427, 379), and the latter two are the length and width of the manual bounding box (*h_i_*, *w_i_*) = (208, 326).

### 3.3. Identification of Defect Types in Workpiece Assembly Based on R-CNN Series Models

**Figure 7:**
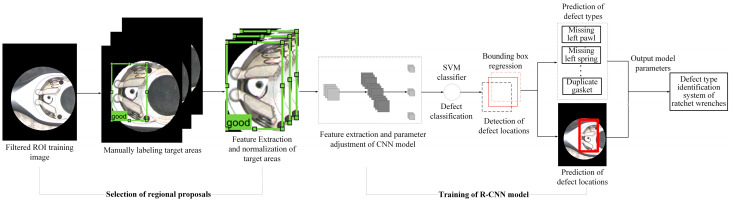
Schematic diagram of a network architecture training procedure using R-CNN model.

After the testing image is filtered and masked to remove noise and background interference, a deep learning network model is used to detect defects. This study uses the R-CNN series network model for defect location and category classification. The R-CNN model is pre-trained on the ImageNet dataset [44] and then fine-tuned for the defect detection tasks. Figure 7 shows a network architecture training procedure using the R-CNN model. The network model can be divided into four steps: selecting object candidate areas, feature extraction of CNN network mode, SVM classifier, and bounding box regression settings [17]. The SVM classification and bounding box regression parts are the preliminary classifications of defects, and the precise location of the defect is further framed to improve detection accuracy.

Generally, four elements constitute a target object: color, size, texture, and shape similarity. Selective search will identify the target object based on the four characteristics in the image area. First, the image segmentation skill is applied to obtain the preliminary segmentation area, and then the hierarchical clustering method is applied to integrate the preliminary segmentation area. The resulting merged area is the candidate region (Region Proposal). After using the selective search method to obtain the candidate region of the original image, the CNN network model is used for feature extraction. In the initial stage, R-CNN mainly uses AlexNet as the leading network to extract features. The size requirement of the input candidate area image is 227 × 227 pixels. The matrix on the image is moved and a convolution operation is performed to extract features.

The principle of the Support Vector Machine (SVM) is to use the principle of minimizing statistical risk to map the corresponding relationship between independent variables and control variables, transforming data from a space of lower dimensionality to one of higher dimensionality, and finding a line in this space to divide the data into two categories and maximize the space of these two categories. SVM can be categorized into three types: linear SVM, non-linear SVM, and inseparable SVM. The type used in this study is the linear SVM.

**Figure 8:**
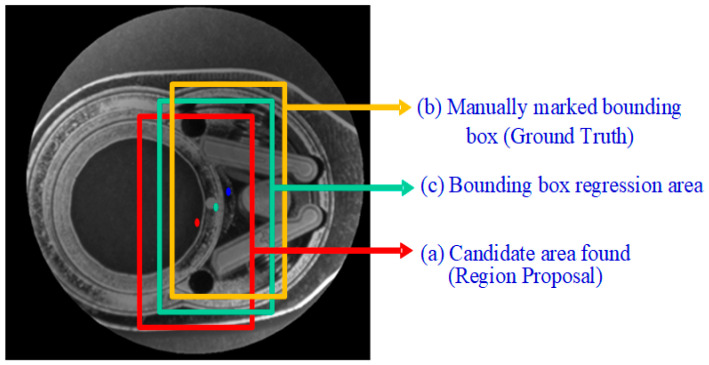
Schematic diagram of bounding box regression principle (three colored dots representing the center points of the corresponding bounding boxes).

**Figure 9:**
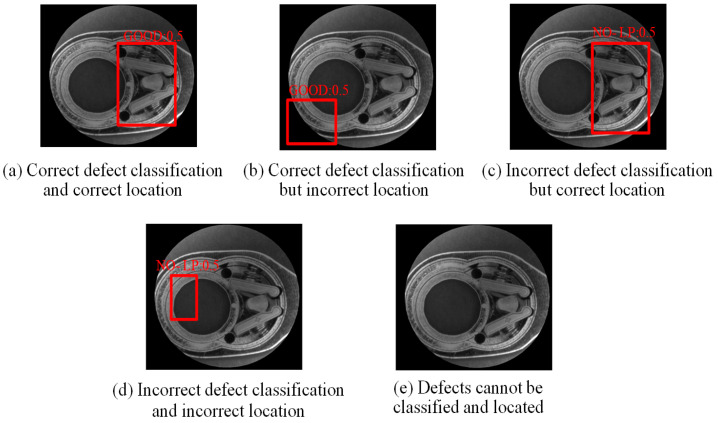
Correct and possible failure results of bounding box regression operations.

Bounding box regression is applied to fine-tune the position of object detection. The concept is shown in Figure 8. Bounding box (a) represents the candidate area found after training by the R-CNN network model (Region Proposal); bounding box (b) represents the manually marked bounding box (ground truth). The goal of bounding box regression is to find the mapping relationship between the two so that the candidate area (a) is mapped to produce a bounding box regression area (c); this area will be closer to the manually marked bounding box (b). The primary purpose of the bounding box regression algorithm is to locate the defect location more accurately. By performing the above operation on the candidate area (a), a bounding box regression area (c) close to the manually marked bounding box (b) is obtained. However, misjudgments may also occur. Figure 9 shows the standard and failure situations that may occur during the bounding box regression calculation process, taking a good product from the first assembly station as an example.

#### 3.3.1. Defect Inspection Based on the R-CNN Model

**Figure 10:**
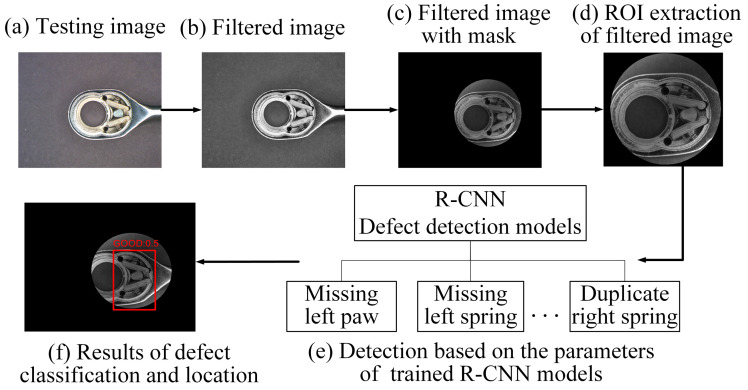
Test procedure for ratchet wrench inspection and identification system using R-CNN network models at the first assembly station.

Suppose only the workpieces at the first assembly station are inspected and classified into various defect types of missing parts after the bounding box regression calculation. In that case, the result obtained is the assembly defect position and classification result of the testing image. There are currently five defect labels for missing parts, three for misplaced parts, four for foreign objects, and two for extra parts at the first assembly station. After training the network models, the testing ratchet wrench image is input into the R-CNN model to identify the defect type in the assembly anomalies. Finally, one should verify whether the output result is the same as the labeled data to evaluate the detection efficiency of this model. Figure 10 shows the testing procedure of the defect-type identification system for the ratchet wrench at the first assembly station.

#### 3.3.2. Defect Inspection Based on the Fast R-CNN Model

**Figure 11:**
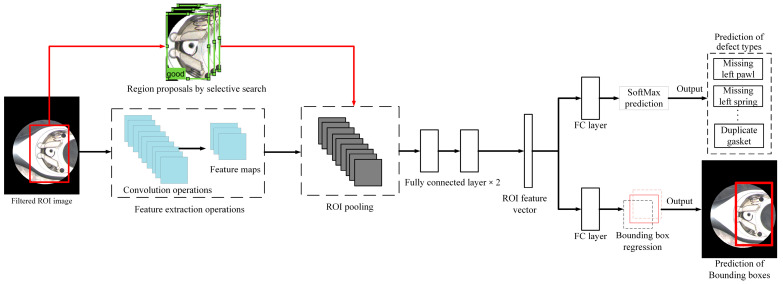
Schematic diagram of fast R-CNN network architecture.

In addition to the R-CNN model, the R-CNN series network models include the Fast R-CNN, Faster R-CNN, and Mask R-CNN models. The combination of the four-step procedures of the R-CNN mode will cause the execution speed of the mode to slow down and cannot handle large datasets. Training an R-CNN model consumes many computing resources, and the processing process requires many procedures and a large amount of storage space; this is its limitation. To decrease the computation time the R-CNN algorithm requires, we can execute CNN only once on each image to obtain all ROI areas instead of processing the number of possible bounding boxes many times. The Fast R-CNN mode processes the CNN only once on each image and then uses a method to perform calculations in multiple regions. When an image is input into CNN, feature mapping graphics will be generated correspondingly. The ROI area can be extracted by using these mapping graphics. Then, a pooling layer is applied to correct all the found ROI areas to the appropriate size. Then, they are forwarded to the fully connected layer for executing classification operations. A Softmax layer generates category results in the backend, and a linear regression layer is applied to create the corresponding bounding box. Therefore, the Fast R-CNN model can simultaneously handle regional feature extraction, classification, bounding box generation, and other procedures. Figure 11 is a flow chart of the application steps of the Fast R-CNN algorithm in this study.

#### 3.3.3. Defect Inspection Based on the Faster R-CNN Model

**Figure 12:**
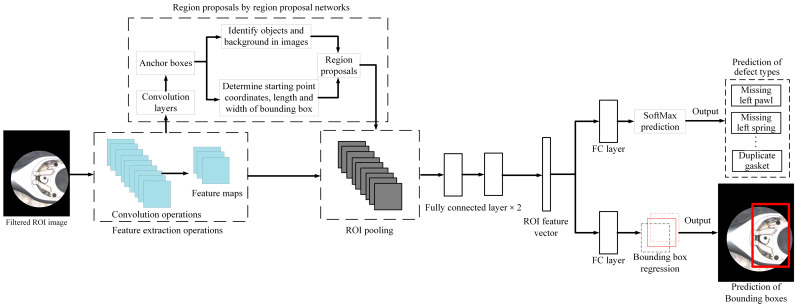
Schematic diagram of faster R-CNN network architecture in this study.

The primary distinction between Faster R-CNN and Fast R-CNN lies in the methodology used for generating the ROI. Fast R-CNN employs a method known as selective search, whereas Faster R-CNN utilizes the Region Proposal Network (RPN) approach. Object proposals can be generated if the input image features are mapped to RPN. Then, the ROI pooling layer is utilized to standardize all the proposals to a uniform size. These adjusted proposals are then forwarded to the fully connected layer to determine the object’s bounding box. The RPN mode employs a sliding window across these feature maps in the Faster R-CNN method. Each window generates many anchor boxes, each with varying shapes and sizes. After RPN processing, the ROI pooling layer can segment each proposal. Following segmentation, each segment contains target objects. The feature map is then relayed to the fully connected layer, classifying the target objects and identifying their bounding boxes. Figure 12 presents a flow diagram illustrating the application of the Faster R-CNN algorithm in this study.

#### 3.3.4. Defect Inspection Based on the Mask R-CNN Model

**Figure 13:**
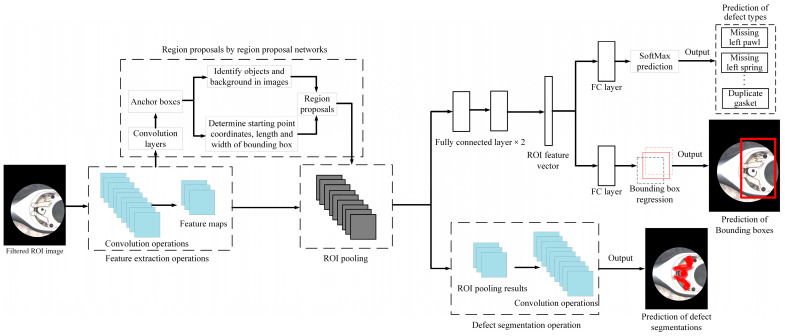
Schematic diagram of Mask R-CNN network architecture in this study.

Mask R-CNN model can find the corresponding bounding box for each target object and mark whether each pixel in the box belongs to the object, achieving a pixel-level instance segmentation effect. It is a two-step architecture. The initial step involves scanning the image to produce candidate frames. The second step is deriving the classified object’s bounding box based on these candidate frames. In addition, the segmentation function is added to the Faster R-CNN architecture to obtain mask results and category predictions. For feature misalignment, Mask R-CNN adds an ROI align layer to record the spatial position accurately and improve the accuracy of the mask position. To maintain spatial structure information, the mask uses a fully connected layer to predict an m*m mask in each ROI. Hence, Mask R-CNN, built upon the Faster R-CNN architecture, yields three outputs for each candidate region: class labels, bounding box adjustments, and object segmentation masks. This mode produces bounding boxes and segmentation masks for each individual object present in the image. Figure 13 depicts a flow diagram outlining this study’s steps in applying the Mask R-CNN algorithm.

### 3.4. Comparison of Defect Detection by R-CNN Series Models

The R-CNN mode algorithm directly converts object detection into a classification problem. However, it uses selective search with high computational complexity to extract candidate frames, and these candidate areas will be repeatedly calculated during feature extraction. Fast R-CNN mode combines the functions of classification and regression. Faster R-CNN proposes a Region Proposal Network (RPN) to find the area to be inspected and replace the original selective search method faster. Mask R-CNN adds a mask prediction function to the Faster R-CNN architecture, which can mark the outline of the target object with a slight increase in calculations. It also adds an ROI align function based on ROI pooling to locate the location of defects more accurately. Therefore, the R-CNN series of models has its correlation and continuity [29].

The Mask R-CNN algorithm performs more detailed individual segmentation of similar objects based on image segmentation, which can achieve streamlined, fast, high-accuracy, and easy-to-use effects. Given that components in industrial precision assemblies are closely assembled, traditional image processing technology faces challenges in managing densely clustered objects. Therefore, this study proposes to apply the algorithms of these R-CNN series models to distinguish closely clustered components and then classify the types of assembly defects and accurately locate the defect locations.

## 4. Experiments and Results

This study summarizes the kinds of assembly anomalies that will occur on the production line and finds the corresponding defect types in the product from these anomaly kinds of assembly. The adopted equipment includes a personal computer (specifications: CPU: Intel^®^ Core™ i7-10700F CPU @2.90GHz, RAM: 32 GB, GPU: NVIDIA GeForce RTX 3070, operating system: Windows 10). We first used optical devices to capture images and the MATLAB (version R2020a) programming language to implement an assembly defect recognition system of ratchet wrenches.

### 4.1. Evaluation Indicators for Classification Efficiency of Defect Types

This study evaluates the detection performance of assembly defect detection and classification and calculates it by comparing the classification results. The classification results are divided into good products and defective products. After judging the ratchet wrench image category, the Type I error (α, the ratio of identifying normal images as defect images) and Type II error (β, the ratio of identifying real defect images as normal images) are calculated. After the preliminary calculation of classification indicators, precision (the proportion of these detected defect images that contain real defects) and recall (1-β, the proportion of correctly identified real defect images among all real defect image sets) are further used to calculate the F1-Score (the measurement of the harmonic mean of precision and recall) and correct classification (CR, the ratio of the number of test images classified to the exact category divided by the total number of test images) indicators to evaluate the performance of the overall detection system. Because this study aims to identify the defect types and their locations, in the defect identification part, the defect type must be correctly classified, and the calculated value of the bounding box reaches more than 50% to be considered correct positioning. If the identification result is of the correct type but the defect location is wrong, it will be judged as a classification error. Similarly, if the category result is wrong but the defect position is correct, it will also be judged as a classification error.

**Figure 14:**
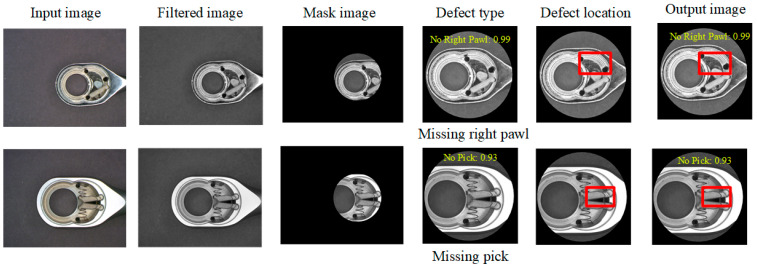
Some detection results of sample experiments.

Figure 14 shows some results of the sample experiments. Most of them can classify the type of defects and locate them with a bounding box (red frame), but a small part of the images still have misjudgments in classification and location. In the R-CNN network model, many model parameters can be adjusted, including learning rate, training batch size, optimizer, training epochs, and the setting of the network model to avoid overfitting. Through the experimental design method [45], a better parameter setting of the R-CNN model is found: learning rate 0.001, training batch size 32, optimizer SGDM, and training epochs 10.

### 4.2. Evaluation and Comparisons

After finding better experimental parameters through small-sample experiments, this study uses large samples for method comparison and performance evaluation. If the first station is taken as an example, then the types and quantities of images in the large-sample experiments in this study are divided into good products and defective products. There are 29 categories of images; each includes 144 training, 36 validation, and 60 testing images. Five network models are used for classification to explore the inspection method’s performance: the R-CNN, Fast R-CNN, Faster R-CNN, Mask R-CNN, and YOLOV4 models. Next, we discuss which model is the most suitable network model for each assembly station in this study.

After selecting the better parameters, we compare the R-CNN series network models. Because the R-CNN series models have multiple evolutions, each model operates differently. Moreover, the inspection efficiency of each model may not be suitable for the assembly station. Therefore, this study conducts large-sample experiments for each assembly station, integrates the types of defects that may occur at the station, and finds the most suitable network model for the assembly station. Table 3, Table 4 and Table 5 summarize the inspection performance indices of various detection models and the traditional manual inspection method at the first, second, and third assembly stations. In various performance indicators, the detection performance of the deep learning models outperforms the manual inspection. Since this study has as many as 28 types of assembly defects with very similar appearances, the accuracy of defect classification is an essential indicator of performance evaluation. According to this rule, the higher the correct classification rate in the three summary tables, the better. The most suitable R-CNN series model for the first station is Faster R-CNN, the second station is Mask R-CNN, and the third station is Fast R-CNN. The R-CNN model requires the most processing time in the training and testing stages, whereas the Faster R-CNN model is the most time-efficient. It is evident from this analysis that Faster R-CNN employs the RPN method for extracting region proposals, thereby significantly reducing the training time.

Regarding object detection technology, the R-CNN and YOLO series are the most commonly used network models and are often compared [33,34]. This study compares the R-CNN series models most suitable for the three corresponding workstations with YOLOV4 models to explore whether the YOLOV4 models are more appropriate for the specific workstations. From Table 3, Table 4 and Table 5, it can be seen that the correct classification rate of the selected R-CNN series model used in each workstation of this study is slightly higher than that of the corresponding YOLOV4 model.

### 4.3. Robustness Analysis of the Proposed Method

This study conducts a sensitivity analysis to evaluate the proposed method’s robustness. This analysis examines the influence of various factors on inspection performance, including the size of the ROI area, the direction of workpiece placement, the quantity of oil stains on the workpiece surface, and the speed of the conveyor belt used for transporting workpieces. The sensitivity analysis has 60 training and 30 testing images for each category of the factors.

#### 4.3.1. The Impact of ROI Area Size on Detection Performance

The purpose of using an ROI mask in this study is to control the detection range to the head of the ratchet wrench during image capture to avoid identification errors caused by background factors. When the ROI mask is smaller, the detected area will be smaller. On the contrary, when the ROI area is larger, the detected area will be more significant. However, the bigger the mask size is, the better. The larger the mask, the more likely it is to be interfered with by noise, such as background or reflection. The smaller the mask, the more likely it is that it will not be able to cover the entire detection area. This study sets five mask sizes for detection system performance analysis. Table 6 shows the detection effects of five mask sizes for this study. It can be seen from the table that a mask radius of 300 has a higher correct classification rate. Therefore, a radius of 300 was selected as the ROI mask for this study.

#### 4.3.2. Effect of Workpiece Placement Direction on Detection Performance

**Figure 15:**
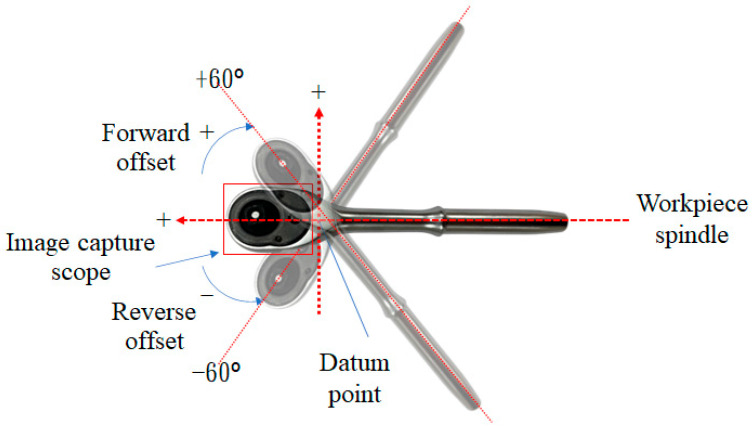
Schematic diagram of various placement directions of the workpieces in this study.

On the conveyor belt during the production process, because the original assembly operation does not have a fixed way of placing workpieces, the placement will vary depending on the assembler. Therefore, this study sets up various ways of placing workpieces to explore how to minimize the impact of artificial differences. Regarding the tilt angle, this study first drew the datum point and the central axis of the workpiece on the carrier plate. Then, the required angles on the carrier plate were drawn to capture images at different angles according to the experiment’s needs. Figure 15 shows the schematic diagram of the various placement directions of the workpieces in this study.

**Figure 16:**
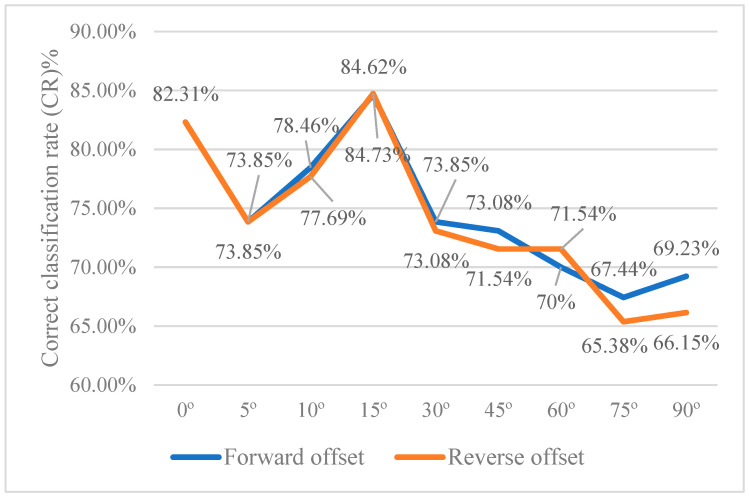
Correct classification rate line chart of ratchet wrenches at various placement angles.

Table 7 shows a comparison table of test effects of ratchet wrenches at different placement angles. It can be seen from the table that the detection effect is the best when the offset angle is ±15°. When the offset angle is larger, the detection effect is worse. As shown in Figure 16, when the offset angle is 0°~±15°, the correct classification rate shows an upward trend. The correct classification rate is the best when the deviation angle is at ±15°. The correct classification rate shows a downward trend when the deviation angle is more significant. Therefore, during the assembly process, an area on the conveyor belt that allows the ratchet wrench to deflect can be planned, and the angle of the area should be controlled within ±15°.

#### 4.3.3. The Impact of the Amount of Oil Stains on the Workpiece Surface on Inspection Performance

In traditional industries, oils such as lubricants are mainly used to lubricate parts. The purpose of lubrication is to reduce the wear and tear between parts, thereby extending the product’s service life. However, the more lubricants are applied, the better the use effect may not be. Too much lubricating oil will affect the use of consumers, and excessive application of lubricating oil will affect the use of the product. Excessive lubricants will cause unnecessary waste to manufacturers and affect the work of assembly personnel, leading to misjudgment.

**Figure 17:**
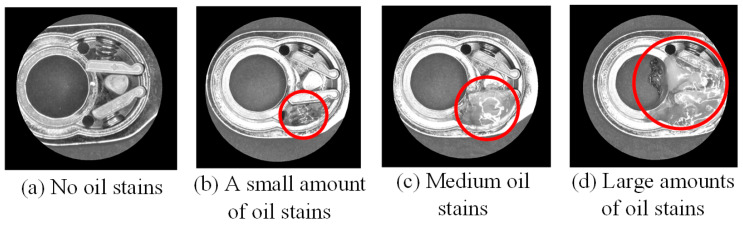
Comparative pictures of the workpieces in the first assembly station after being coated with different lubricant levels (red circles) and then preprocessed and masked.

In this study, lubricating oil must be applied when the torque head is installed at the second station during the assembly process. To explore the impact of lubricating oil on detection effects, this section is based on the assembly operations of the first and second stations. In the definition of lubricating oil application amount, the appropriate oil stain amount interval is defined based on the area ratio of the ROI area of the first and second stations and the degree of lubricating oil application. This study describes it as a small amount when the lubricating oil application area accounts for less than or equal to 10% of the ROI area, a medium amount when 10 to 30% is used, and a large amount greater than 30%. The first and second stations were shot with different lubricant levels in this section. The images, being captured and preprocessed, are shown in Figure 17.

The comparison of detection effects between the first and second stations applying different levels of lubricating oil is shown in Table 8. It can be seen from the table that the more lubricating oil is used at the first station, the lower the correct classification rate is. It is worth noting that the second station must apply lubricating oil during the assembly process. When the amount of lubricating oil is reduced to less than 10%, its detection effects will be better than those without lubricating oil.

#### 4.3.4. The Impact of Conveyor Belt Speed for Conveying Workpieces on Inspection Performance

**Figure 18:**
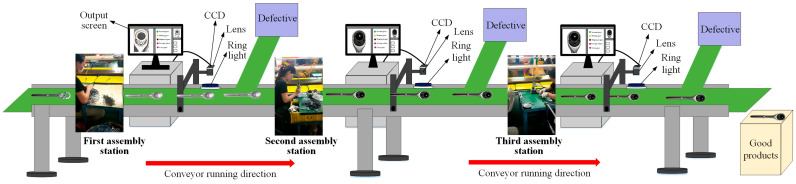
Schematic diagram of the operation of the ratchet wrench dynamic visual inspection system.

**Figure 19:**
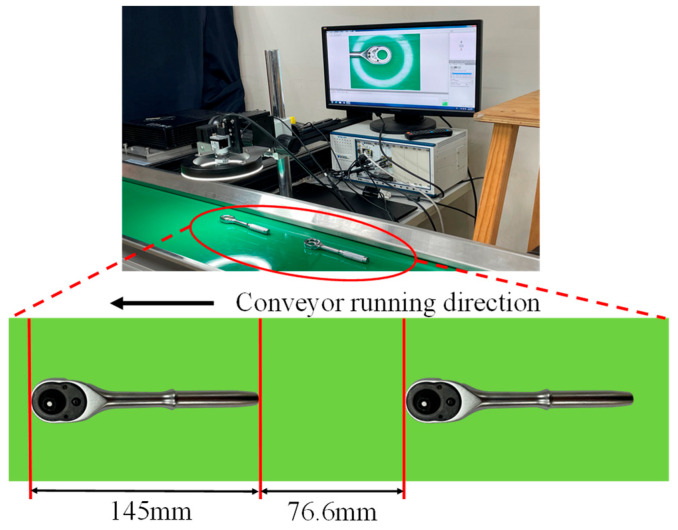
Hardware configuration diagram of ratchet wrench dynamic visual detection system.

Material handling between ratchet wrench assembly stations mainly relies on conveyors. In this study, a dynamic visual inspection system is set up between assembly stations to better meet the industry’s needs in inspecting assembly defects. This system includes the conveyor for the work-in-process handling of ratchet wrenches, the CCD with lens and fixed frame for capturing images, the computer vision equipment that can process and output the captured images, and the transmission line to connect the CCD to the computer vision equipment for inspection work. Figure 18 shows the schematic diagram of the operation of the ratchet wrench dynamic visual inspection system. The actual installation part is shown in Figure 19. The equipment used includes personal computers (specifications): CPU: Intel(R) Core(TM) i7-10700F CPU @2.90GHz; RAM: 32 GB; GPU: NVIDIA Geforce RTX 3070; operating system: Windows 10; 1.3 million pixel color CCD. The length and width of the conveyor belt are 100 × 35 cm, the size of the ratchet wrench is 14.5 cm, and the distance between workpieces is 7.66 cm.

**Figure 20:**
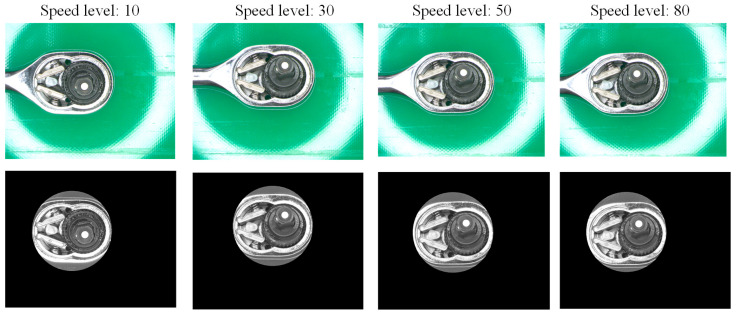
Original and preprocessed images captured at different conveyor speed settings in the dynamic visual inspection system.

The dynamic visual inspection system sequentially transports the ratchet wrenches to the CCD via the conveyor belt for photography. The speed of the conveyor belt can be adjusted according to the user’s needs. However, the faster the conveyor belt speed is set, the higher the detection effect may not be. If the belt speed is too fast, it may cause afterimages or blur in the captured images. If it is too slow, it will affect production efficiency. This study adjusts the speed of the conveyor belt and compares the detection efficiency under different speed settings. After analysis, the appropriate speed is selected as the setting for the ratchet wrench dynamic visual inspection system. The corresponding speeds are shown in Table 9. The original and preprocessed images captured at different conveyor speed settings are shown in Figure 20.

**Figure 21:**
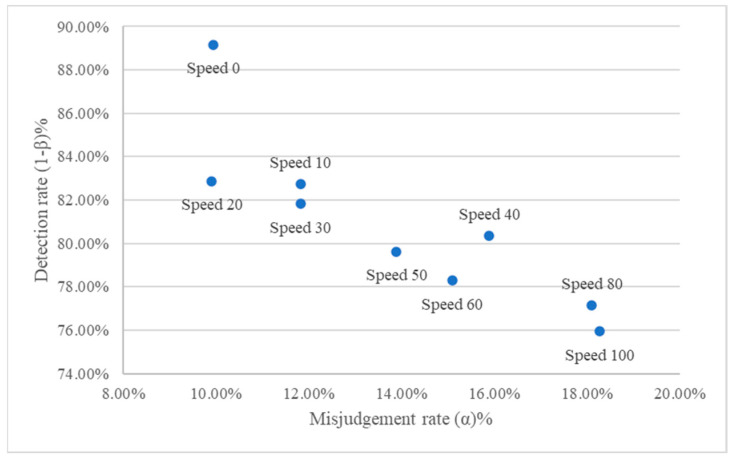
ROC plot of ratchet wrench dynamic visual inspection system under different conveying speed settings.

**Figure 22:**
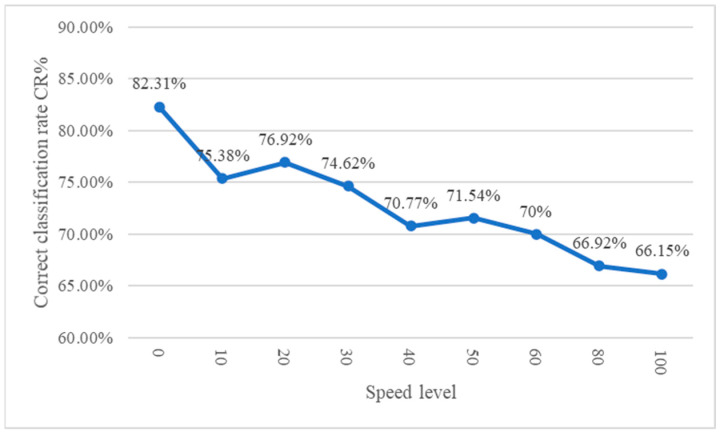
Correct classification rate curve of ratchet wrench dynamic visual inspection system under different conveying speed settings.

The detection performance when setting different conveyor speeds in this study is shown in Table 10. As can be seen from the table, when the conveyor speed is set in the range of 10 to 30; the detection rate is above 81%, but when the conveyor belt speed exceeds 30, the detection rate decreases. It can be seen from the receiver operating characteristic (ROC) plot [46] and the correct classification rate curve in Figure 21 and Figure 22 that the detection effect of speed 20 is better and more suitable for this study’s dynamic visual detection system. In the correct classification rate part, when the conveyor belt speed level is between 10 and 30, there is a better correct classification rate, and the maximum value is 76.92% when the conveyor belt speed level is 20.

Other impacts of automated inspection on the workforce and data privacy should also be considered. Automated inspection systems can significantly improve efficiency and accuracy in quality control processes. However, it is important to recognize that implementing these systems could lead to changes in the workforce. For instance, some roles may become obsolete, while others, particularly those involving the operation and maintenance of automated systems, may become more prominent. It is crucial to emphasize retraining and upskilling the workforce to adapt to these changes. Furthermore, automated inspection systems generate and process a large amount of data, raising valid concerns about data privacy. Robust data management policies are essential to ensure the privacy and security of the data. This could include measures such as anonymizing data, implementing strict access controls, and ensuring compliance with relevant data protection regulations.

## 5. Concluding Remarks

This study takes the wrench assembly process as an example to explore the automated detection of hand tool assembly defects in the precision assembly process. Using traditional machine vision and classification techniques to detect assembled parts with tightly clustered and embedded properties is challenging. This study proposes inspection technology and system development for classifying assembly defects and determining the defect locations based on computer vision and deep learning techniques to facilitate the production of industrial assembly products.

This study samples the work-in-process from three assembly stations in the ratchet wrench assembly process, investigates 28 common types of assembly defects in the assembly operation, and uses CCD to capture sample images of various assembly defects for experiments. First, the captured images are filtered to eliminate surface reflection noise from the workpiece; then, a circular mask is given at the assembly position to extract the ROI area; next, the filtered ROI images are used to create a defect-type label set using manual annotation; after this, the R-CNN series network models are used for object feature extraction and classification; finally, it is compared with other object detection network models to identify the model with the better performance. The experimental results show that, if each station uses the best model for defect inspection, it can effectively detect and classify defects. The average defect detection rate (1-β) of each station is 92.64%, the average misjudgment rate (α) is 6.68%, and the average correct classification rate (CR) is 88.03%. This study also investigates the impact of diverse factors on inspection performance to assess the robustness of the proposed methodology. Further research could explore examining existing approaches to search for the most efficient and effective method for the proposed application, applying the transfer learning method to establish assembly defect detection models for other types of hand tools to expand their multiple applications, and using advanced analytics and machine learning techniques to incorporate and interpret multi-sensor data, etc.

## Figures and Tables

**Table 1:** Summary table of possible assembly anomaly kinds that may occur during ratchet wrench assembly.

Assembly Anomaly Types	Description	Examples	Images
Missing components	This refers to the situation where a component that should have been included in the assembly is absent.	The left claw is missing when assembling the workpiece	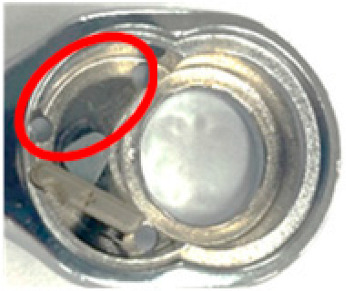
Misplaced components	This refers to components that, while present, are not situated in their correct position within the assembly. Such as being installed in an incorrect location, orientation, or alignment.	When assembling the workpiece, the left and right pawls are in the wrong position.	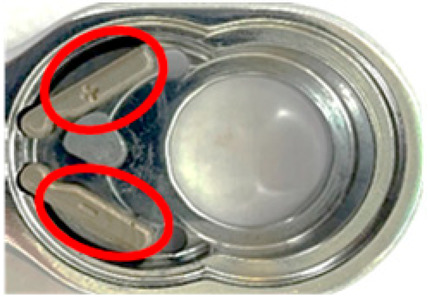
Foreign objects	This refers to instances where an object, which is not a part of the assembly, is discovered within the workpiece.	When assembling a workpiece, insert parts that do not belong to the workpiece.	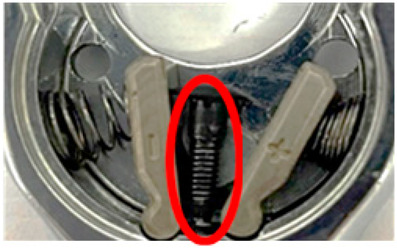
Extra components	This refers to instances where additional components, which should not be part of the assembly, are present.	When assembling the workpiece, add an extra washer to the assembled workpiece.	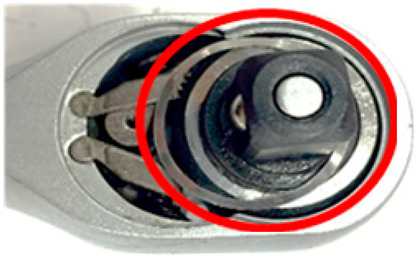

**Table 2:** Data table of mask and image area used in this study (unit: pixel).

Image Size	1,310,720 (1024 × 1280)				
Mask Circle Center	(800, 540)				
Mask radius	250	300	350	400	450
Mask area	196,250	282,600	384,650	502,400	635,850
Ratio of mask to image area	14.97%	21.56%	29.35%	38.33%	48.51%

**Table 3:** Summary table of the inspection performance indices of various detection models at the first assembly station.

Models	R-CNN	Fast R-CNN	Faster R-CNN	Mask R-CNN	YOLOv4	Manual Inspection
Misjudgment rate (α)%	20.45	18.56	8.44	8.70	8.70	22.75
Detection rate (1-β, Recall)%	82.63	83.38	88.75	88.49	86.19	76.13
Precision (P)%	80.16	81.79	91.32	91.05	90.84	72.52
Correct classification rate (CR)%	70.00	71.78	82.89	82.44	80.44	62.24
F1-Score %	81.38	82.58	90.01	89.75	88.45	74.28
Testing time (s)	36.3	1.71	0.80	1.11	0.61	8.59

**Table 4:** Summary table of the inspection performance indices of various detection models at the second assembly station.

Models	R-CNN	Fast R-CNN	Faster R-CNN	Mask R-CNN	YOLOv4	Manual Inspection
Misjudgment rate (α)%	17.20	16.46	10.27	9.94	10.61	20.07
Detection rate (1-β, Recall)%	84.39	85.44	89.12	89.16	88.79	77.98
Precision (P)%	83.07	83.85	89.67	89.97	89.33	73.83
Correct classification rate (CR)%	73.59	74.87	82.05	82.31	81.54	64.85
F1-Score %	83.73	84.64	89.39	89.56	89.06	75.85
Testing time (s)	35.84	1.62	0.76	0.92	0.64	6.63

**Table 5:** Summary table of the inspection performance indices of various detection models at the third assembly station.

Models	R-CNN	Fast R-CNN	Faster R-CNN	Mask R-CNN	YOLOv4	Manual Inspection
Misjudgment rate (α)%	8.62	1.67	5.08	3.39	0.00	9.97
Detection rate (1-β, Recall)%	94.83	100.00	96.61	94.92	98.33	88.69
Precision (P)%	91.67	98.36	95.00	96.55	100.00	86.25
Correct classification rate (CR)%	91.11	98.89	94.44	94.44	98.89	83.58
F1-Score %	93.22	99.17	95.80	95.73	99.16	87.45
Testing time (s)	11.04	1.89	0.49	0.90	1.80	3.06

**Table 6:** Comparison of detection performance indices using different mask sizes.

Mask radius (unit: pixel)	250	300	350	400	450
Misjudgment rate (α)%	37.50	21.50	23.19	16.44	17.35
Detection rate (1-β, Recall)%	64.50	71.50	57.97	63.47	59.36
Precision (P)%	63.24	76.88	71.43	79.43	77.38
Correct classification rate (CR)%	51.33	64.33	55.00	61.33	57.67
F1-Score %	63.86	74.09	64.00	70.56	67.18

**Table 7:** Comparison table of test effects of ratchet wrenches at different placement angles.

Offset Direction	Forward (Clockwise)							
Offset Level	Small (<30°)			Medium (30°~60°)			Large (>60°)	
Offset Degree	+5°	+10°	+15°	+30°	+45°	+60°	+75°	+90°
Misjudgment rate (α)%	15.09	11.93	7.08	16.04	18.45	19.42	21.78	21.78
Detection rate (1-β, Recall)%	83.02	86.24	89.38	83.96	84.47	81.55	80.20	82.18
Precision (P)%	84.62	87.85	92.66	83.96	82.08	80.77	78.64	79.05
Correct classification rate (CR)%	73.85	78.46	84.62	73.85	73.08	70.00	67.44	69.23
F1-Score %	83.81	87.04	90.99	83.96	83.25	81.16	79.41	80.58
Offset direction	Reverse (counterclockwise)							
Offset level	Small (<30°)			Medium (30°~60°)			Large (>60°)	
Offset degree	−5°	−10°	−15°	−30°	−45°	−60°	−75°	−90°
Misjudgment rate (α)%	12.84	12.04	6.96	14.81	18.10	16.98	21.78	24.00
Detection rate (1-β, Recall)%	81.65	85.19	89.57	82.41	82.86	82.08	77.23	80.00
Precision (P)%	86.41	87.62	92.79	84.76	82.08	82.86	78.00	76.92
Correct classification rate (CR)%	73.85	77.69	84.73	73.08	71.54	71.54	65.38	66.15
F1-Score %	83.96	86.38	91.15	83.57	82.46	82.46	77.61	78.43

**Table 8:** Comparison table of detection effects of applying various amounts of lubricating oil on workpieces.

Station	Degree of Lubricating Oil Applied	Small Amount (<10%)	Medium Amount (10~30%)	Large Amount (>30%)
First station	Misjudgment rate (α)%	11.93	19.05	24.24
	Detection rate (1-β, Recall)%	86.24	82.86	73.74
	Precision (P)%	87.85	81.31	75.26
	Correct classification rate (CR)%	78.46	71.43	61.54
	F1-Score %	87.04	82.08	74.49
Second station	Misjudgment rate (α)%	14.02	19.42	24.24
	Detection rate (1-β, Recall)%	86.92	82.52	73.74
	Precision (P)%	86.11	80.95	75.26
	Correct classification rate (CR)%	77.69	70.77	61.54
	F1-Score %	86.51	81.73	74.49

**Table 9:** Image detection efficiency of different conveyor speed settings.

Conveyor speed level (cm/s)	10 (3.49)	20 (7.92)	30 (11.17)	40 (14.80)
Shooting interval (s)	6.80	3.63	2.24	1.6
Inspection efficiency (pieces/min)	10	22	31	41
Conveyor speed level (cm/s)	50 (20.75)	60 (25.85)	80 (28.77)	100 (28.77)
Shooting interval (s)	0.89	0.91	0.84	0.83
Inspection efficiency (pieces/min)	57	70	78	78

**Table 10:** Inspection effect comparison table of ratchet wrench dynamic visual inspection system at different conveyor speed settings.

Conveyor speed level (cm/s)	10 (3.49)	20 (7.92)	30 (11.17)	40 (14.80)
Misjudgment rate (α)%	11.82	9.91	11.82	15.89
Detection rate (1-β, Recall)%	82.73	82.88	81.82	80.37
Precision (P)%	87.50	89.32	87.38	83.50
Correct classification rate (CR)%	75.38	76.92	74.62	70.77
F1-Score %	85.05	85.98	84.51	81.90
Conveyor speed level (cm/s)	50 (20.75)	60 (25.85)	80 (28.77)	100 (28.77)
Misjudgment rate (α)%	13.89	15.09	18.10	18.27
Detection rate (1-β, Recall)%	79.63	78.30	77.14	75.96
Precision (P)%	85.15	83.84	81.00	80.61
Correct classification rate (CR)%	71.54	70.00	66.92	66.15
F1-Score %	82.30	80.98	79.02	78.22

## Data Availability

The data will be made available on request.
